# Evaluation of a hybrid asynchronous-synchronous delivery model for family intervention in psychosis: the psychosis REACH program

**DOI:** 10.3389/fdgth.2026.1662969

**Published:** 2026-07-21

**Authors:** Sarah L. Kopelovich, Jennifer Blank, Akansha Vaswani-Bye, Victoria Shepard, Helen Teresa Buckland, Kate Hardy, Douglas Turkington

**Affiliations:** 1 Department of Psychiatry and Behavioral Sciences, University of Washington School of Medicine, Seattle, WA, United States; 2 Department of Psychiatry and Behavioral Sciences, Stanford University School of Medicine, Palo Alto, CA, United States; 3 Cumbria Northumberland Tyne and Wear NHS Foundation Trust, Sunderland, Tyne and Wear, United Kingdom

**Keywords:** bichronous learning, caregivers, family intervention for psychosis, hybrid digital training, skills training

## Abstract

**Introduction:**

The uptake of Family Interventions for psychosis (FIp) in routine settings has been limited across high-, middle-, and low-income countries due to geographic, systemic, and socioeconomic barriers. Psychosis REACH is a FIp that addresses these barriers by using a direct-to-family model co-facilitated by licensed mental health practitioners with expertise in Cognitive Behavioral Therapy for psychosis and family members with lived experience. While prior delivery relied on in-person training, this approach does not adequately address structural access challenges. The aim of this investigation was to evaluate caregiver outcomes of a hybrid asynchronous-synchronous version of Psychosis REACH consisting of (a) an online course, (b) a live virtual skills workshop, and (c) follow-along skills coaching from a trained family peer (Psychosis REACH Family Ambassador; pRFA).

**Materials and methods:**

Psychosis REACH was delivered via a hybrid digital format to 275 caregivers of individuals with psychosis, the majority of whom were parents (61.2%). We evaluated feasibility, acceptability, and participant characteristics, along with outcomes including depression, anxiety, caregiver burden, expressed emotion, attitudes toward psychosis, and self-perceived CBT skillfulness at post-training and 4-month follow-up.

**Results:**

Both asynchronous and synchronous components were rated as highly feasible and acceptable. Nearly all participants completed the online course; 85% went on to complete the virtual workshop, of which 66.7% endorsed intention to follow-up with a pRFA. Participants demonstrated significant improvements across multiple domains, including reduced psychiatric symptoms, caregiver burden, stigmatizing attitudes, and expressed emotion, along with increased confidence in CBT-informed skills.

**Discussion:**

Findings suggest that this hybrid digital training is both acceptable and feasible, offering a promising approach for disseminating evidence-based family support in psychosis. Clinical and educational outcomes were comparable to in-person delivery, underscoring its potential to reduce access barriers for some caregivers. Future research should prioritize randomized trials and active engagement of diverse and underserved families.

## 1 Introduction

Evidence from recent systematic reviews and network meta-analyses confirms that psychosocial and psychological interventions—including family interventions and cognitive behavioral therapy (CBT)—significantly reduce relapse risk in psychotic disorders (1–3), yet these interventions are difficult to access through clinic-based care settings (4). Psychosis Recovery by Enabling Adult Carers at Home (Psychosis REACH) is a family intervention for psychosis (FIp) that aims to bridge the practice gap observed in routine clinical settings by providing psychoeducation and cognitive behavioral skills training directly to families caring for a loved one with psychosis (5, 6). To enhance applicability, the intervention was intentionally designed as a transdiagnostic family intervention applicable across illness stages. The intervention covers three domains of content: [1] recovery-oriented psychosis psychoeducation, [2] cognitive behavioral techniques for psychosis that can be implemented by non-clinicians, and [3] the role of caregiver self-care in psychosis recovery. Content is delivered by both clinical experts (licensed psychiatrists and psychologists with specialized training in CBT for psychosis), and trained family peers called Psychosis REACH Family Ambassadors (pRFAs) (7, 8). Because the intervention is grounded in Cognitive Behavioral Therapy for psychosis (CBTp), Psychosis REACH creates synergies between cognitive behavioral interventions provided to individuals experiencing psychosis by their clinical team and community-based caregiving provided by the client's natural or residential support team. The coactive approach between clinic-based and community-based supports, referred to in the global public health literature as *task sharing,* can facilitate the reciprocal transfer of therapeutic skills across the healthcare setting and the home environment (9, 10). Furthermore, caregivers' self-application of CBT skills and other forms of self-care normalize and model CBT for the individual experiencing psychosis and the family system.

Previous research suggests that the in-person Psychosis REACH training significantly enhanced caregivers' knowledge and skills and reduced caregivers' depressive and anxiety symptoms (5). Moreover, results suggest that the intervention was associated with an enhanced recovery orientation, reductions in self-perceived caregiver burden, and reductions in caregivers' expressed emotion–a characteristic of the family milieu associated with high levels of criticism, hostility, or anxiety that is positively correlated with symptom relapse in a wide range of mental disorders, including psychosis spectrum disorders (11). Caregivers reported significantly improved mastery over the five CBTp-informed skill domains in which they were trained. All intervention effects held at 4-months follow-up with one exception: caregivers reported decreased self-efficacy in delivering CBTp-informed skills to their loved ones at follow-up. The depreciation in skill mastery is consistent with the broader practice facilitation literature, which emphasizes the importance of rehearsal with feedback during the skill acquisition and generalization phases (12, 13). These findings suggest an important area of refinement in the approach to training families. In addition, the in-person training did not remedy inequities in access to evidence-based interventions among socially, geographically, and economically disadvantaged families, nor did it enhance access to FIp among non-maternal natural supports.

*Hybrid digital training* (alternatively referred to in the literature as bichronous learning) utilizes technology to facilitate both asynchronous and synchronous pedagogic activities (14). The learner-centric method enhances access through multimodal on-demand activities that can accommodate diverse learning styles through a range of instructional practices. Bichronous training has traditionally been applied to and studied in workplace and academic settings (15, 16) and little is known about the acceptability, feasibility, or learner outcomes associated with psychological intervention training for lay people. A recent systematic review found that caregiver interventions for psychosis that were entirely digitally delivered can improve caregiver stress, expressed emotion, and parental self-efficacy (17) although the included interventions were predominantly education and did not include facilitated skill rehearsal.

To support broader access to Psychosis REACH and to capitalize on the affordances of a direct-to-family intervention, the authors developed a hybrid digital training program. The hybrid Psychosis REACH program consists of an asynchronous distance learning course, where didactic and reflective content is delivered by mental health specialists, and a synchronous skills works, which is co-delivered by a psychiatrist and/or clinical psychologist with expertise in psychosis, pRFAs, and a clinician moderator (a mental health professional responsible for facilitating the training, annotating the role plays, and moderating the question-and-answer exchanges following each role play). We evaluated the acceptability and feasibility of this hybridized synchronous-asynchronous delivery format and explored whether the blended training could achieve caregiver outcomes comparable to those previously reported for the live in-person training.

## 2 Materials and methods

This investigation represents a program evaluation of a hybrid digitally-delivered FIp (Psychosis REACH). The protocol was reviewed by the University of Washington Institutional Review Board and determined to qualify for Exempt Category 2 status under federal regulations governing research involving survey and interview procedures. All participants provided informed consent prior to participation. Given the direct-to-consumer format, we focused on outcomes most relevant to end users—acceptability (satisfaction with and perceived relevance of the intervention), feasibility (extent to which the intervention can be successfully carried out), and intentions to engage—as well as preliminary effectiveness outcomes for caregivers. This focus was selected because early evaluation of a modified delivery model requires understanding whether caregivers find the intervention usable, relevant, and worth engaging in before broader implementation questions can be meaningfully tested. Additionally, because caregivers often manage competing demands related to their loved one's needs, employment, other family responsibilities, and advocacy activities, interventions that are difficult to access or perceived as not immediately useful are unlikely to be completed even when interest is high. Therefore, assessing feasibility and acceptability was essential to determining whether this delivery model could address known barriers to FIp engagement.

### 2.1 Development of blended Psychosis REACH training

The Psychosis REACH training described previously consisted of a combination of live (synchronous) didactics, self-reflection opportunities, and case-based role plays facilitated by clinical experts in a large-group format. The impetus to revise the training was based on caregiver feedback from post-training evaluations, including requests for greater flexibility in pacing, opportunities to revisit complex content, and additional skill-practice support, and the desire to enhance caregiver outcomes by better aligning with best practices in remote instruction (19). Specifically, caregivers indicated desire for more flexible timing and pacing of didactic content, opportunities to revisit complex material, and extended skill practice opportunities beyond the initial training. Conceptual revisions to the training approach were spearheaded by authors SLK, AVB, and HTB. Technical revisions were guided by a professional in adult learning theory and instructional design with an emphasis on maximizing engagement, retention, and accessibility within the blended learning environment. Revisions to the course were guided by the relevant elements of the IDEAS framework for teaching online; namely, *Inclusion*, which refers to providing opportunities to ensure that all participating caregivers feel they are welcomed and belong in the course; *Design*, which focuses on incorporating instructional materials, activities, resources, and assessments that are aligned with learning outcomes; and *Engagement*, which promotes caregiver interactions and community building across learning activities (20).

Didactic content was transitioned to a self-paced online distance learning course hosted on a secure web-based learning management system aligned to Web Content Accessibility Guidelines [WCAG (21);]. Content intended to enhance caregiver proficiency across the skill domains was organized into discrete learning modules with an accompanying assessment activity to better capture knowledge acquisition. Guided by Quality Matters (QM) standards, participating caregivers completed an orientation module to acclimate themselves to the learning management system, course navigation, structure and pacing, technical support, and the sequence of the online course prior to interacting with course modules. Upon completion of the modules, caregivers received access to an online community board and resource repository. During the live virtual workshop, caregivers were encouraged to identify their geographic location to cultivate connection, provided with a brief review of the five skill domains that were introduced in the online course, and guided in a moderated demonstration and post-demonstration discussion of each of the five CBTp skill domains. Caregivers were provided multiple opportunities to ask questions during the workshop, which were answered live and/or in the meeting chat. Frequently Asked Questions were archived and made accessible to caregivers in the learning management system. Finally, caregivers were encouraged to connect with a pRFA following the workshop for continued behavioral rehearsal with feedback as well as for emotional support.

### 2.2 Recruitment

Three international training cohorts, each enrolling up to 100 caregivers, participated between May 2021 and February 2022. Analyses included participants from all three cohorts to characterize user experience, engagement, and caregiver outcomes in a naturalistic, real-world setting. Because this evaluation was intended to characterize user experience, engagement, and caregiver outcomes among participants in a globally disseminated Psychosis REACH training program, data were analyzed across the full sample of participating caregivers rather than by country. Each training cycle was advertised on online mental health forums and by word of mouth by a local Family and Caregiver Advisory Board, with a specific emphasis on notifying Black, Indigenous, and People of Color (BIPOC)-serving mental health advocacy and activist organizations. Consenting caregivers were invited to complete three research surveys: prior to the Psychosis REACH online course (T1), immediately following the Psychosis REACH live workshop (T2), and 4-months after the live workshop (T3). Participating caregivers received a $20 gift card subsequent to their participation. Participation was voluntary and did not impact registrants' exposure to any element of the training.

### 2.3 Measures

We analyzed three sets of outcomes: caregiver demographics, caregiver perceptions of the blended training, and learning and psychiatric outcomes across the three timepoints (pre-training, post-training, and 4-month follow-up).

#### 2.3.1 Caregiver demographics

Caregivers self-administered a demographic questionnaire in which they were asked to self-report their race, gender, country of residence, age, relationship to their loved one with psychosis, background information on their loved one (e.g., diagnosis of record, time since diagnosis, mental health services received), length of time caring for their loved one, and extent of their involvement in their loved one's mental health care, if applicable. We also queried about lifetime history of receiving CBTp (loved one) or FIp (self).

#### 2.3.2 Caregiver perceptions

Consistent with implementation evaluation frameworks (18), we examined several indicators of the program's caregiver-facing outcomes—specifically perceptions of acceptability and feasibility, as well as engagement patterns relative to uptake. Within this context, acceptability refers to caregivers' satisfaction with and perceived relevance of the training; feasibility refers to the practicality of engaging with the digital components; and engagement patterns refer to participation in and completion of the digital training components. To further assess feasibility, we also calculated the proportion of caregivers who completed each of the two main components of the hybrid digital training (the online course and the live workshop). Next, we asked caregivers to rate their intention to follow-up with a pRFA from 1 (*very unlikely*) to 3 (*highly likely*) after the online course and after the live event. Finally, given that advocacy is a tertiary goal of Psychosis REACH training, we were interested in capturing the extent to which caregivers felt empowered to engage in advocating for greater availability of CBTp (“*After completing Psychosis REACH, how motivated are you to advocate for the greater availability of CBT for psychosis*;” responses were rated on a 5-point Likert scale ranging from 1 (*very unmotivated*) to 5 (*very motivated)*. These acceptability and feasibility items were developed by the research team based on constructs from Proctor et al.'s implementation outcomes framework (18) and online learning evaluation principles (19, 20). Items were reviewed for face validity by the Family and Caregiver Advisory Board and piloted informally with a small group of caregivers prior to full use; however, formal psychometric validation was not undertaken in this program evaluation.

#### 2.3.3 Learning and psychiatric outcomes

To enable *post hoc* comparison of the hybridized virtual format to the preliminary investigation of Psychosis REACH, we adopted the same battery [see Kopelovich et al. (5), for descriptions of each measure] and adhered to the same administration schedule. To reduce burden on participating caregivers, we substituted the 66-item Experience of Caregiving Inventory (ECI) with the 19-item Brief Experience of Caregiving Inventory (BECI (22); to assess positive and negative aspects of the caregiving experience. The BECI has demonstrated good internal consistency (Cronbach's *α* = 0.84) and convergent validity with the ECI (22), supporting comparability of constructs measured, though direct comparison of absolute scores across studies is not possible. However, both versions measure caregiver burden and allow for evaluation of change over time within each study. The direction and pattern of change can be meaningfully compared across studies, even if absolute values cannot. To reduce the risk of self-selection bias, we surveyed all caregivers partaking in Psychosis REACH, regardless of their progression through the multicomponent training.

### 2.4 Statistical analysis

Analyses were descriptive and exploratory, consistent with the aims of a program evaluation. Because of the nature of the investigation, the sample consisted of caregivers who self-enrolled in the training program and opted to complete evaluations at each of the three timepoints. To ensure manageable training cohort sizes and access to PRFA resources following training for all enrolled caregivers, we chose to restrict training cohorts to 100 caregivers. Due to the exploratory nature of this program evaluation and pre-determined cohort sizes, *a priori* power analyses were not conducted. Sample size was therefore determined by programmatic capacity rather than formal power targets. For transparency, *post hoc* power estimates were calculated for the completed analyses and indicated power of.96 or above; these values should be interpreted cautiously given the known limitations of *post hoc* power analysis. Longitudinal changes were analyzed with a paired sample *t*-test when only two repeated measures were present (e.g., BECI) or a one-way repeated-measures within subjects analysis of variance (ANOVA) for measures present across all three time points (PAS, HADS, FAS, CBTp-informed skills).

Normality of data was assessed using the Shapiro–Wilk test and by examining skewness and kurtosis coefficients, box plots, histograms, and Q-Q plots. Based on examination of box plots, no extreme scores were observed. Data was found to be normally distributed unless otherwise reported. Statistically significant omnibus *F*-tests indicated that scores were not the same across each timepoint within the full sample and were further examined with *post hoc* Bonferroni-adjusted comparisons to evaluate changes between timepoints. Listwise deletion was used to manage missing data. For all analyses, alpha was set at *p* < .05. Effect sizes were calculated using partial eta squared, except when indicated by analysis. We adopted Cohen's heuristic of.01,.06 and.14 partial eta squared values corresponding to small, medium, and large magnitudes, respectively (23) All analyses were completed using SPSS version 26.

## 3 Results

### 3.1 Caregiver characteristics

Of those who registered for Psychosis REACH training (*N* = 303) between May 2021 and February 2022, 275 agreed to participate in the surveys (90.75%). Caregiver demographics are reported in Table 1. We did not detect significant differences across training cohorts in self-identified race, gender, age, marital status, or education. Caregivers who consented to the survey disproportionately identified as non-Hispanic and monoracial white (84.7%), female (82.1%), married (66.3%), and highly educated (80.5% reported a bachelor's degree or higher). Caregivers originated from 34 US states (95.3%); four Canadian provinces (1.5%); as well as San Jose, Costa Rica (.4%); Wellington, New Zealand (.4%); Bihar, India (.4%); and the United Kingdom (.4%). Caregivers were most likely to be the parent of the individual with psychosis (61.2%); 78.1% reported their loved one's principal diagnosis was a primary psychotic disorder, followed by bipolar disorder (9.8%), major depressive disorder (3.4%), or a major neurocognitive disorder (2.9%). The remainder (5.7%) were unsure. Secondary diagnoses included anxiety, major depressive disorder, substance use disorder, and post-traumatic stress disorder (PTSD). Loved ones were most commonly diagnosed within the past 5 years (57.2%); the remainder were diagnosed between 6 and 40 years prior to the training. Sixty-four caregivers (23.3%) reported that their loved one had not received treatment for psychosis, whereas the majority reported that they were either receiving consistent care (57.1%) or were engaged inconsistently (11.6%). To assess the extent to which caregivers could use the skills they would be learning in Psychosis REACH, caregivers were asked if they were currently in weekly contact with their loved one. Roughly half (44%; *n* = 121) endorsed this item; however, item data were missing or invalid for a large proportion of our sample (62.9%, *n* = 173). Finally, we were interested in ascertaining lifetime history of indicated clinical interventions for psychosis. According to our respondents, 22.5% of caregivers' loved ones had received a course of CBTp; 21.2% endorsed receiving a FIp. Conversely, 25.3% reported that neither they nor their loved one had received any intervention related to the psychotic illness.

**Table 1:** Caregiver demographics.

Characteristic	M	SD	Range
Age	57.26	11.84	24–88
	**N**	**Valid %**	
Gender	273	100	
Female	225	82.4	
Male	46	16.8	
Non-Binary	2	0.7	
Race^a^	275	100	
African American	13	4.7	
Asian American	10	3.6	
White	233	84.7	
Latino/Hispanic	21	7.6	
Native American	3	1.1	
Biracial	24	8.8	
Prefer not to disclose	5	1.8	
Level of Education	246	100	
High school graduate or less	5	2.0	
Some college/other training	23	9.3	
Associates degree	20	8.1	
Bachelor's degree	71	28.9	
Graduate or similar	23	9.3	
Master's degree	76	30.9	
Doctoral degree	28	11.4	
Marital Status	246	100	
Single	30	12.2	
Married	163	66.3	
Separated or divorced	43	17.5	
Widowed	7	2.8	
Other	3	1.2	
Relationship to loved one with psychosis	178	100	
Parent	109	61.2	
Child	38	21.3	
Sibling	17	9.6	
Significant Other	6	3.4	
Grandchild	2	1.1	
Friend	4	2.2	
Other	2	1.1	
Time since diagnosis	159	100	
Less than 1 year	21	13.2	
1–5 years	70	44.0	
6–10 years	35	22.0	
11–20 years	23	14.5	
21–30 years	8	5.0	
31–40 years	2	1.3	
Prior exposure to FIp	235	100	
Yes	120	51.1	
No	94	40.0	
Not sure	21	8.9	

a Participants were able to select more than one race.

FI*p*, family intervention for psychosis.

### 3.2 Caregiver perceptions

#### 3.2.1 Acceptability of blended learning

Among caregivers who completed the post-course survey (*n* = 177), 92.1% agreed or strongly agreed that the information presented in the online course was personally relevant. Most respondents (88.2%) agreed or strongly agreed that it provided information that was relevant to both their culture and community. We assessed acceptability following the live virtual workshop; most respondents (88.6%) agreed or strongly agreed that the synchronous workshop presented information that was personally relevant. Similarly, most caregivers (84.7%) agreed or strongly agreed that the synchronous workshop was relevant to their community.

#### 3.2.2 Feasibility of blended learning

Nearly all caregivers (94.4%) completed the online modules. Eighty-five percent of caregivers who completed the online course also attended the workshop. Among those who did not, the most common reasons for not attending the workshop were work (25%) and scheduling conflicts (15%). Almost all respondents (91.6%) either agreed or strongly agreed that they were able to manage the technology used during the online course. Finally, following the live virtual workshop, 81.6% of caregivers either agreed or strongly agreed that they were able to use technology during the live event.

#### 3.2.3 Intentions

Two-thirds (66.7%) of caregivers indicated they were highly likely to connect with a pRFA to rehearse CBTp-informed skills taught in Psychosis REACH. We attempted to capture actual contact with a pRFA 12-months post-workshop in a random sample of 100 Psychosis REACH participants. Only 16 caregivers responded to the survey request. The majority of those who did respond denied contact with a pRFA (87.5%). Finally, respondents overwhelmingly intended to advocate for greater availability of CBTp following the training (*M* = 4.7, *SD* = .71, mode = 5).

### 3.3 Learning and psychiatric outcomes

Descriptive statistics of outcome measures across all times points are summarized in Table 2^1^ and time series analyses are described below.

**Table 2:** Summary of learning and psychiatric outcomes.

Outcome measure	Pre-training M (SD)	Post-training M (SD)	Follow-up M (SD)
Caregiver Burden (BECI)	*n* = 185	–	*n* = 98
	38.07 (10.77)		30.96 (10.11)
Psychosis Attitudes (PAS)	*n* = 220	*n* = 119	*n* = 101
	77.59 (14.96)	96.96 (14.33)	96.17 (13.52)
Anxiety (HADS)	*n* = 226	*n* = 127	*n* = 101
	9.57 (4.14)	7.69 (3.48)	7.52 (3.97)
Depression (HADS)	*n* = 227	*n* = 123	*n* = 105
	6.34 (4.04)	4.40 (3.28)	3.99 (3.37)
Expressed Emotion (FAS)	*n* = 198	*n* = 119	*n* = 97
	46.73 (20.81)	38.24 (19.55)	35.11 (18.57)
CBTp-Informed Skill Mastery	*n* = 222	*n* = 123	*n* = 98
	19.62 (8.62)	27.36 (7.50)	28.09 (7.41)

BECI, brief experience of caregiver inventory; PAS, psychosis attitude scale; HADS, hospital anxiety and depression scale; FAS, family attitude scale.

#### 3.3.1 Caregiving experiences

A dependent means *t-*test comparing self-reported caregiver burden at pre-training and 4-month follow-up on the BECI (22) revealed a significant reduction in caregiver burden at follow-up [*M_T_*_1_ = 37.14, *SD_T_*_1_ = 10.76; *M_T_*_3_ = 30.78, *SD_T_*_3_ = 9.45; *t*(76) = 6.04, *p* < .001, *d* = 0.689 (.95 CI = 0.438, 0.935)].

#### 3.3.2 Psychosis attitudes

We compared caregivers' scores on the Psychosis Attitudes Scale (24) at each of the three time points using a one-way repeated measures ANOVA. Mauchly's test of sphericity indicated that the assumption of sphericity was met for the three-way interaction [*χ*^2^(2) = 0.081, *p* = .96]. We found a statistically significant main effect for time *F*(2,144) = 115.37, *p* < .001, partial *η*^2^ = .62, with pairwise comparisons revealing a statistically significant increase in prosocial attitudes toward psychosis from pre-training (*M_T_*_1_ *=* 79.66, *SD_T_*_1_ *=* 14.49) to post-training (*M_T_*_2_ *=* 98.36, *SD_T_*_2_ *=* 13.63, *p* < .001, *d* = −1.58) and sustained at 4-month follow-up (*M_T_*_3_ *=* 97.07, *SD_T_*_3_ *=* 12.84 *p* < .001, *d* = −1.43).

#### 3.3.3 Psychiatric symptoms

The Shapiro–Wilk test revealed a violation of the assumption of normality for T1 (*W* *=* .947, *p* *=* .003), T2 (*W* *=* .928, *p* *<* .001), and T3 (*W* *=* .886, *p* *<* .001) of the depression subscale of the Hospital Anxiety and Depression Scale [HADS (25);]. Upon further examination, skewness and kurtosis were within acceptable ranges for each time point (skewness ranged between |.58| and |1.24|; kurtosis ranged between |.18| and |1.84|) and no extreme scores were identified. For this reason and ANOVAs relative robustness to violations of this assumption (26), a one-way repeated measures ANOVA was employed. Mauchly's test of sphericity indicated that the assumption of sphericity was met for the three-way interaction, *χ*^2^(2) = 1.114, *p* = .573. We found a statistically significant main effect for time *F*(2,158) = 10.05, *p* < .001, partial *η*^2^=.11, with pairwise comparisons revealing a decrease in self-reported depression from pre-training (*M_T_*_1_ = 5.36, *SD_T_*_1_ = 3.73) to post-training (*M_T_*_2_ *=* 4.39, *SD_T_*_2_ = 3.29 *p* = .005) and sustained at 4-month follow-up (*M_T_*_3_ *=* 4.09, *SD_T_*_3_ = 3.59, *p* < .001).

The Shapiro–Wilk test revealed a violation of the assumption of normality for T3 HADS Anxiety scores (*W* *=* .957, *p* *=* .011), but not for T1 (*W* *=* .980, *p* *=* .252) or T2 HADS Anxiety scores (*W* *=* .976, *p* = .154). Further inspection of T3 data revealed skewness and kurtosis to be within the acceptable range (.73 and.41, respectively) and no extreme scores were identified. As such, a one-way repeated measures ANOVA was deemed appropriate. Mauchly's test of sphericity indicated that the assumption of sphericity was met for the three-way interaction, *χ*^2^(2) = 3.473, *p* = .176. A statistically significant main effect was observed for time *F*(2,158) = 10.797, *p* < .001, partial *η*^2^ = .12, with pairwise comparisons revealing decreased anxiety from pre-training (*M_T_*_1_ *=* 9.05, *SD_T_*_1_ = 4.05) to post-training (*M_T_*_2_ *=* 7.60, *SD_T_*_2_ = 3.36, *p* < .001), sustained at 4-month follow-up (*M_T_*_3_ *=* 7.56, *SD_T_*_3_ = 4.05, *p* = .001).

#### 3.3.4 Expressed emotion

The Shapiro–Wilk test revealed a violation of the assumption of normality for T1 (*W* *=* .955, *p* *=* .015) and T2 (*W* *=* .958, *p* *=* .023) of the Family Attitudes Scale (FAS (27); scores, but not for T3 (*W* *=* .969, *p* *=* .085). Examination of the data revealed deviations to be minimum, with skewness and kurtosis to be within acceptable ranges (skewness ranged between |.64| and |.71|; kurtosis ranged between |.22| and |.33|) and no extreme scores identified. Therefore, a one-way repeated measures ANOVA was employed. Mauchly's test of sphericity indicated that the assumption of sphericity was met for the three-way interaction, *χ*^2^(2) = 1.944, *p* = .378. We found a statistically significant main effect for time *F*(2,134) = 8.84, *p* < .001, partial *η*^2^ = .12, with pairwise comparisons revealing a statistically significant decrease in frequency of hostility and emotional over-involvement (*M_T_*_1_ *=* 41.34, *SD_T_*_1_ = 20.43*; M_T_*_2_ *=* 35.60, *SD_T_*_2_ = 18.42, *p* < .001) that were sustained at follow-up (*M_T_*_3_ *=* 36.12, *SD_T_*_1_ = 19.34, *p* = .006).

#### 3.3.5 Mastery of CBT-informed skills

Results of the Shapiro–Wilk test revealed the data to be relatively normally distributed (*p* > .05). Mauchly's test of sphericity indicated that the assumption of sphericity had been violated for the three-way interaction, *χ*^2^(2) = 25.845, *p* < .001; we therefore applied the Greenhouse-Geisser correction on the one-way repeated measures ANOVA comparing caregivers' CBTp skill mastery across all time points. We observed a statistically significant main effect for time [*F*(1.537,146) = 68.83, *p* < .001, partial *η*^2^ = .49], with pairwise comparisons revealing a significant increase in applying CBTp-informed skills following the training (*M_T_*_1_ *=* 18.86, *SD_T_*_1_ = 9.02; *M_T_*_2_ *=* 27.32, *SD_T_*_2_ = 7.07, *p* < .001) and sustained 4 months later (*M_T_*_3_ *=* 28.23, *SD_T_*_3_ = 6.98, *p* < .001).

## 4 Discussion

Psychosis REACH is a CBT-based FIp that is designed to be delivered directly to families and caregivers who are navigating a loved one's psychotic illness. In doing so, the intervention leverages both clinical experts and experts by experience called pRFAs to support families in learning information and skills that can bolster psychosis recovery. In recognition of the limitations of the original model—namely, that in-person delivery is a rate limiting factor for its uptake—we set out to improve upon the original delivery model by applying best practices for online learning to support a comprehensive hybrid training delivery that could enhance inclusion, design principles, and engagement. Such an approach has been successfully deployed for other behavioral health priorities that similarly benefit from family education and skills training (28). We sought to ascertain the feasibility and acceptability of the hybrid training approach and to explore the assumption that the adapted modality could replicate in-person outcomes previously reported (5). The current investigation benefited from a naturalistic setting, large sample size, and guiding implementation and online instructional frameworks.

While prior exposure to FIp in the current sample were higher than rates reported in earlier national audits (4), changes in family intervention and coordinated specialty care availability over the past decade may partially account for these differences. In particular, the substantial expansion of coordinated specialty care programs following national dissemination efforts such as RAISE and related initiatives has likely increased opportunities for family intervention participation relative to the period captured by earlier national audits. Additional explanations may relate to characteristics of the current sample, which was disproportionately drawn from socially advantaged backgrounds, more likely to have a loved one engaged in treatment, and more likely to have been referred by a family advocacy organization or behavioral health agency. Nevertheless, a substantial proportion of families remain unable to access FIp through clinic-based delivery platforms. Interventions that bypass clinic-based administration, such as Psychosis REACH, may help address this gap but mist make a concerted effort to reach families for whom clinic-based services are least accessible, as structural disadvantages, socioeconomic adversities, and concerns about the applicability of the intervention may continue to impede equitable reach. In practice, this may require partnerships with community-based organizations serving underrepresented families, provision of technology support such as tablets or subsidized internet access, offering training in community-based settings and multiple languages, and developing targeted peer outreach led by Family Ambassadors from diverse backgrounds. Future studies should prospectively evaluate whether these strategies improve access, engagement, and retention among underserved caregivers.

Results suggest that FIp delivered through a fully remote hybrid digital approach are acceptable and feasible to caregivers supporting a loved one with psychosis. This finding is consistent with a systematic review indicating that digital interventions for caregivers of individuals experiencing a first episode of psychosis can reduce stress and expressed emotion and improve self-efficacy (17). Caregivers reported that the content across the online course and live workshop were both personally and culturally relevant, which may be a reflection of the significant inclusion of pRFAs with lived experience in the intervention development and delivery. In addition, nearly all caregivers completed the online course and most went on to attend the workshop, suggesting that the sequenced structure and technical requirements of the hybrid digital training were not prohibitive for our sample. Interestingly, a higher proportion of participants denied technology difficulty with the online course compared to the live Zoom-based workshop, implicating both the technical accessibility of the online component for our sample and the need for technical support during live virtual trainings. Finally, two-thirds of participants endorsed intentions to connect with a pRFA following both the online course and live virtual workshop, yet most participants who responded to our inquiry 12 months after their training had not done so. Although these data must be interpreted with extreme caution given the low response rate to this inquiry, future research should explore the hindrances to pRFA follow-up and investigate the effectiveness of different solutions to increase post-training practice opportunities. Nevertheless, the virtual co-presence of a caregiver community, the opportunity to observe a pRFA sharing their personal narrative, and the balance of being able to learn didactic content at their own pace followed by live demonstrations of real-life scenarios may have improved encoding, salience, and retention, leading to improved caregiver outcomes. Future investigations should identify mechanisms of change (29). Nearly all participants indicated their intention to advocate for CBTp as a result of their training experience, suggesting that educating families about indicated interventions for psychotic disorders may translate to grassroots advocacy. Given the historical impact that families have had in driving policy and payment changes in the US mental healthcare system (30) and given the poor accessibility of CBTp in the US (31), this finding engenders hope in the potential for Psychosis REACH to impact the accessibility of both FIp and CBTp.

Our findings related to caregiver burnout, psychosis attitudes, caregiver symptoms of anxiety and depression, and expressed emotion were comparable to findings reported by Kopelovich et al. (5) despite the hybridized training approach. We observed changes in the expected direction across all measures from baseline to post-workshop that were sustained at 4-month follow-up. While the current investigation used the abbreviated version of the ECI to assess caregiver burnout, which limits direct comparison across studies for this measure, reductions in burden were observed across both, with the current investigation yielding a larger effect size. Furthermore, in contrast to the investigation of the in-person administration of Psychosis REACH, hybrid participants reported sustained gains in self-perceived CBTp informed skills mastery, suggesting that skill retention may be improved in the blended training approach. This finding is likely attributable to the fact that bichronous participation allows caregivers to self-pace and revisit material presented in the online course. Reduction in expressed emotion and, relatedly, caregivers' own symptoms of anxiety and depression, and enhanced mastery of CBTp-informed strategies have implications for improving the relational dynamics between family members and may have downstream implications for relapse prevention among individuals with psychosis (5, 32, 33).

### 4.1 Limitations and future directions

Our evaluation suggests that a blended FIp training modality consisting of a self-paced online course and a live virtual skills workshop can enhance geographic access and may preserve caregiver outcomes relative to prior in-person delivery, although stronger conclusions regarding effectiveness and reach require more rigorous study designs. That said, several limitations to the current investigation are noteworthy. A primary limitation of this effort was its naturalistic design. In the absence of a randomized controlled trial, our data provide a signal that our blended training approach is effective in promoting greater caregiver well-being and reduced expressed emotion and psychosis stigma. Combined with prior quantitative and qualitative findings (8), our data provide cause for further investigation into the effectiveness of Psychosis REACH in addressing both caregiver and loved one mental health outcomes. A fully powered trial should also explore potential covariates. While Stiles and colleagues (34) did not find support for duration of illness as a significant moderator of caregiver outcomes, caregiver and loved one characteristics along with previous exposure to treatment should be explored as potential moderators of intervention response. Future randomized clinical trials should investigate clinical and functional outcomes among the loved ones with psychosis as well as qualitative perceptions of the loved one to better understand the potential impact of Psychosis REACH on multiple members of the family system. Furthermore, participants represented a range of countries with differing healthcare systems, cultural understandings of psychosis, and family support resources. As a program evaluation, the goal was to characterize outcomes among the overall population of Psychosis REACH participants rather than examine country-specific differences. Future studies with larger samples are needed to investigate how cultural and healthcare system factors may influence implementation, engagement, and outcomes.

Beyond effectiveness research, future studies should also evaluate implementation directly, including adoption across service settings, staffing and supervision requirements for pRFAs, sustainability across diverse contexts, integration with clinical services, and implementation strategies that can improve equitable engagement across diverse caregiver populations. Econometric analyses of Psychosis REACH compared to comparable clinic-based FIp can help to establish potential cost benefits, cost effectiveness, and cost utility of an extramural FIp like Psychosis REACH. Such data are critical to future implementation-focused work that is needed to remediate the poor penetration and sustainment of FIp to-date. Established implementation frameworks such as RE-AIM or CFIR may help structure this next phase of research.

Similar to the in-person training cohort reported by Kopelovich and colleagues (5), our participants were most commonly white, highly educated mothers of adults who were diagnosed with a psychotic spectrum disorder with the last 5 years. Thus, the shift to virtual training alone, even when combined with efforts to engage diverse families, was insufficient in this sample to demonstrate equitable reach across gender, relational, racial, ethnic, and socioeconomic groups. Nevertheless, our sample was substantially more geographically diverse than the original sample, suggesting that the transition to a blended training model can enhance geographic reach of FIp. Efforts are currently underway to enhance outreach to diverse communities and stakeholders, explore cultural relevance among racially and ethnically diverse communities, incorporate more culturally responsive materials, and engage in enhanced coalition-building.

While it is reasonable to hypothesize a dose-response relationship, we were unable to reliably assess the extent to which participants engaged with each component, and thus, how the degree of engagement with the various components of the hybrid digital training may influence outcomes. This was especially true regarding participants' engagement with a pRFA following completion of the hybrid training. Our attempt to capture engagement with a pRFA 12-month post-workshop in a subset of participants suggests that few participants contacted pRFAs for further support. While we are unable to draw any firm conclusions from this data due to the low response rate, the low level of uptake of pRFA coaching that was reported by respondents may suggest that a more accessible pipeline from training to community-based supports is needed. Similarly, because a large proportion of participants did not respond to our inquiry related to the frequency of contact with their loved one, we were unable to analyze between group differences among those who endorsed weekly contact and those who did not. Future research should investigate the relationship between concurrent FIp and naturalistic opportunities to apply new information and skills training both with and without follow along support.

## 5 Conclusion

Psychosis REACH participants overwhelmingly found the hybrid digital training approach to be both acceptable and feasible, suggesting that it may offer a promising method for delivering FIp. The benefits of participation were comparable to previous reports of in-person training. Randomized controlled trials coupled with efforts to engage more diverse and underserved families must be undertaken to establish the effectiveness of Psychosis REACH and support its equitable dissemination.

## Data Availability

The datasets presented in this article are not publicly available because they contain information collected as part of a program evaluation involving caregivers of individuals with psychosis, and public sharing could compromise participant confidentiality. The study was supported through philanthropic funding and the Washington State Health Care Authority and was not subject to funder data-sharing requirements. De-identified data may be made available by the corresponding author upon reasonable request for purposes of verifying the reported findings or conducting secondary analyses, subject to institutional review, applicable ethical and legal requirements, and execution of an appropriate data use agreement.

## Footnotes

^1^Table 2 includes descriptive statistics (i.e., number of observations, mean, standard deviation) for all observations across pre-training, post-training, and 4-month follow-up. As such, means and standard deviations may differ slightly from those reported in the text due to procedures employed to account for missing data.
